# Ivacaftor Therapy in CF Patients: Single Center Experience

**DOI:** 10.1155/2014/947923

**Published:** 2014-10-22

**Authors:** Pritish Mondal, Amber Loyson, Jorge Lascano, Satyanarayan Hegde

**Affiliations:** ^1^Department of Pediatrics, University of Florida, 1600 SW Archer Road, Suite HD 604, P.O. Box 100296, Gainesville, FL 32610, USA; ^2^Division of Pulmonary, Critical Care and Sleep Medicine, Department of Medicine, University of Florida, 1600 SW Archer Road, P.O. Box 100225, Gainesville, FL 32610, USA; ^3^Division of Pediatric Pulmonary Medicine, Department of Pediatrics, University of Florida, 1600 SW Archer Road, P.O. Box 100296, Gainesville, FL 32610, USA

## Abstract

Ivacaftor is the first novel cystic fibrosis pharmaceutical that acts at the molecular level to potentiate cystic fibrosis transmembrane conductance regulator (CFTR) function and was first approved for clinical use in 2012. We are sharing our single center experience of five patients: four from pediatric age group and one adult patient. All patients had both subjective and objective improvements in their health. Despite established lung disease, our patients had significant improvement in both their FEV1 (forced expiratory volume in 1 second) and FEF_25–75_ and BMI (body mass index). Larger studies demonstrated only 6.7% improvement in mean FEV1 after starting Ivacaftor therapy but their patient population had normal lung function to begin with. In contrast our case series demonstrates that, in patients with established lung disease and diminished lung function, Ivacaftor can be expected to result in much higher recovery in lung function. Mean FEV1 improved by 35% in our case series. Ivacaftor is extremely expensive, costing $300,000 per patient per year requiring lifelong therapy, hence requiring prior authorizations from most third-party payers in the USA. The knowledge shared from our experience will be useful for other clinicians to petition healthcare policymakers on behalf of their patients.

## 1. Introduction

Cystic fibrosis (CF) is the most common life-threatening autosomal recessive disease in the USA [1]. There is a wide variety of gene sequences in cystic fibrosis and more than 1900 CF mutations have been identified [2]. Although daily airway clearance therapies and pancreatic enzyme replacement are keys to CF management, the introduction of Pulmozyme and the introduction of inhaled antibiotics were two milestones in CF management and have become routine care in established lung disease in CF patients. However, there was no known medication that could correct this disease and its underlying molecular defect until recently. Ivacaftor, also known as Kalydeco, is the first marketed drug which restores the function of the cystic fibrosis transmembrane conductance regulator (CFTR). It was approved by the United States Food and Drug Administration (FDA) in January 2012 for treating cystic fibrosis patients with G551D mutation, older than six years. It has shown to be effective as evidenced by reduction in the sweat chloride content of the subjects as well as by the improvement in FEV1 up to 10 percent [3].

Due to its prohibitive cost (approximately $300,000/patient/year), Ivacaftor therapy requires prior authorization from insurers. These insurers often rely on published postmarketing data in order to add newer therapies to their approved lists. In premarketing clinical trials, Ivacaftor demonstrated sustained improvement in FEV1, body mass index (BMI) and the frequency of pulmonary exacerbations [3]. Recently Rowe et al. [4] published a large prospective study with six-month followup of Ivacaftor therapy demonstrating modest but significant improvement in lung function in relatively healthy patients. We are sharing our single center experience of five patients at the University of Florida Pediatric and Adult Cystic Fibrosis Program. We have followed up our patients for 15 months.

## 2. Methodology

The University of Florida CF database was queried to identify patients with specific G551D mutation. There were a total of 7 patients with at least one copy of the G551D mutation. Of these, 5 patients were above the age of 6 years who qualified for and were started on Ivacaftor therapy. A retrospective chart review was performed. Pulmonary function results, BMI measurements, radiology, and sputum culture results were extracted. Data collected before and after starting Ivacaftor were directly compared. Three longitudinal data sets of FEV1, FEF_25–75_, and BMI values were analyzed separately using R software by fitting mixed-effects models to each one of them. “Lmer” command was implemented in the lme4 package of the R library.

## 3. Results

In this direct comparison, by verbal survey, all patients reported subjective improvement in their health status. None of the patients experienced any adverse effects. There were four pediatric patients and one adult patient who were followed up for at least 15 months after starting Ivacaftor therapy (Table 1).

**Figure 1:**
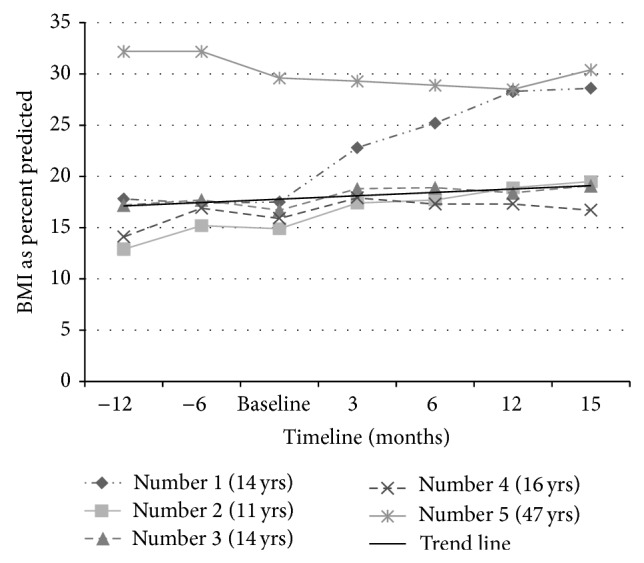
BMI trend. There was a significant overall improvement in BMI (*P* < 0.01). The adult patient (patient number 5) was obese to start with who lost some weight to a healthier BMI.

**Figure 2:**
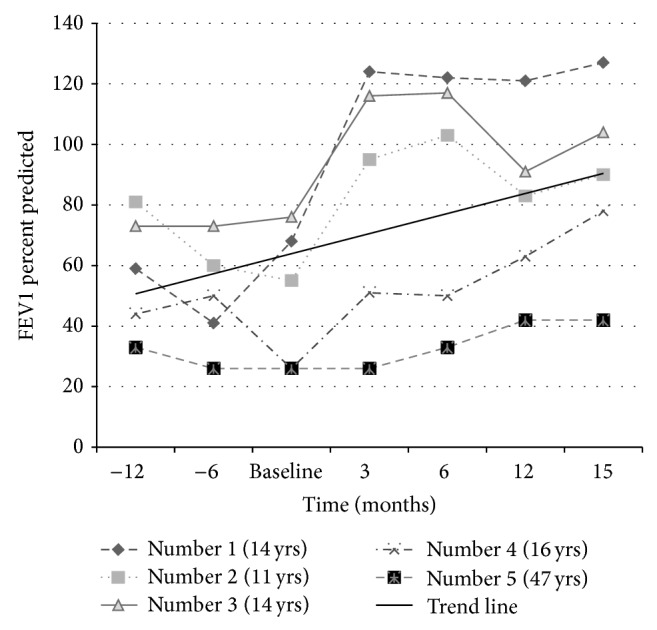
FEV1 trend. There was significant overall improvement (*P* < 0.01) in FEV1, however more so in the younger patients. Patient number 5 (46-year-old female) who already had established bilateral bronchiectasis had a modest improvement. Patient number 4, a 14-year-old male who also had bilateral diffuse bronchiectasis and multiple pulmonary exacerbations prior to starting Ivacaftor, demonstrated significant improvement in his FEV1 presumably due to decreased mucus impaction in the airways (see chest CT scans in Figure 4).

**Figure 3:**
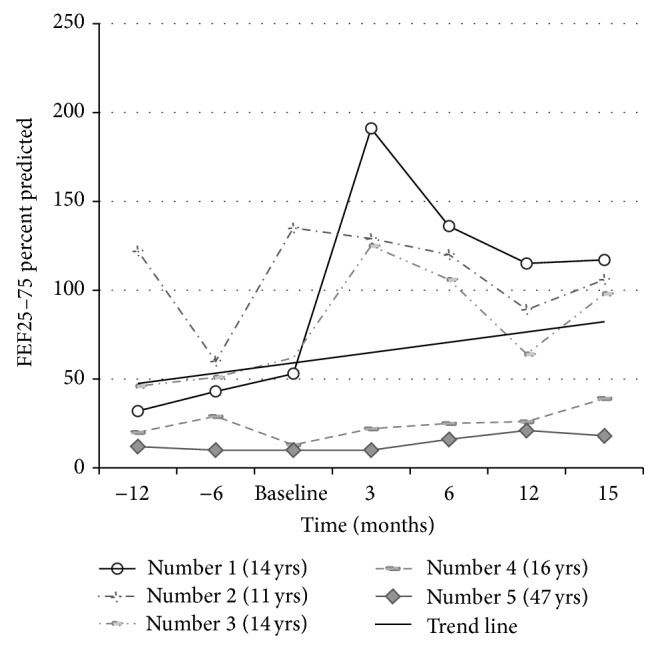
FEF_25–75_ trend. Similar to trend in FEV1 (see Figure 2), there was significant improvement in FEF 25–75% (*P* < 0.01). The adult patient (number 5) had only modest improvement suggesting that the patient already had irreversible lung damage.

The mean BMI among pediatric patients was 16.2 kg/m^2^ at baseline which improved to 20.9 kg/m^2^ at 15 months (Figure 1). The mean FEV1 changed from 56.2 to 99.7 percent predicted (Figure 2) and the mean FEF_25–75_ from 40.2 to 90 percent predicted (Figure 3). The adult patient was a 45-year-old female who had been previously diagnosed with advanced lung disease when Ivacaftor was started. Her FEV1 was 23% at 3 months after beginning of the therapy, which changed to 42% at 15 months after Ivacaftor. Her BMI was 29.6 kg/m^2^ at baseline which did not change significantly with time. Our results yield a *P* value of less than 0.01 for all the three data sets analyzed separately for FEV1, FEF_25–75_, and BMI. The adult patient was known to be pancreatic sufficient and her obesity was a contributing factor to her diabetes mellitus. Her HbA_1c_ improved from 7.4% to 6.6% without changing any insulin regimen after Ivacaftor was started. She had one hospitalization for CF exacerbation in the year before starting Ivacaftor though she did not require any hospitalization after Ivacaftor therapy.

**Figure 4:**
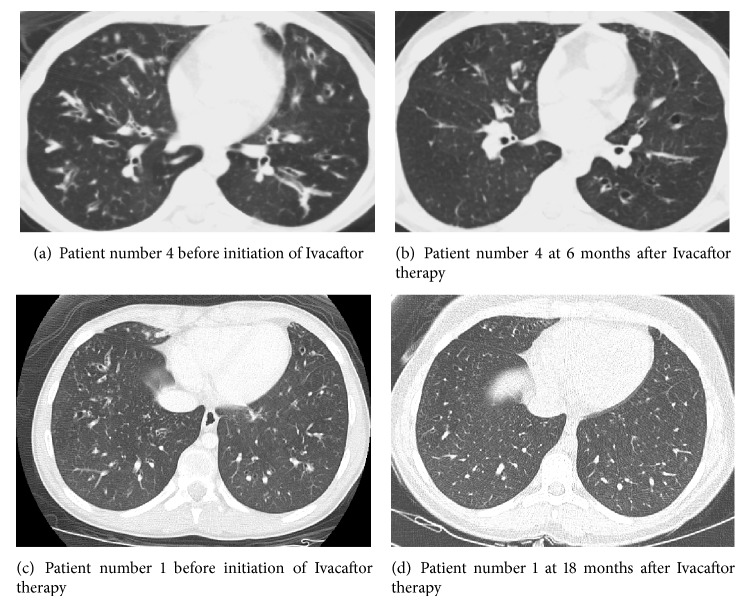
CT of the chest before and after Ivacaftor. Two representative chest CT slices of patient number 4 ((a) and (b), 16-year-old male) at the same anatomic region before and after Ivacaftor therapy. They demonstrate decreased mucus impaction in the latter scan despite the continued presence of bronchiectasis. Decreased mucus impaction probably explains improved lung function (Figures 2 and 3) despite irreversible nature of bronchiectasis. In the bottom panels ((c) and (d)) are the chest CT of another patient (14-year-old female) who already had established bronchiectasis in the peripheral airways before Ivacaftor (c) which has been resolved 18 months after Ivacaftor therapy (d).

Ivacaftor also had an impact on the sputum microbiology (Table 1). Two of our patients had methicillin resistant* Staphylococcus aureus* (MRSA) before, which could not be isolated after Ivacaftor therapy. One of them, who used to grow nonmucoid pseudomonas before in bronchoalveolar lavage (BAL), is now growing only methicillin-sensitive* Staphylococcus aureus* (MSSA), since we have started Ivacaftor. In two patients, where pre- and post-chest CT scans were available, there was significant improvement in the radiology findings, which showed decreased mucus impaction, improved bronchial wall thickening, and partial reversal of bronchiectasis despite established lung disease (Figure 4). During the same period, his FEV1 has improved from 26% predicted to 50% after Ivacaftor therapy. The vitamin D level did not change significantly.

## 4. Discussion

As mentioned previously, Ivacaftor is an expensive medication costing about $300,000 per year per patient, and patients require lifelong therapy [5]. When an exorbitantly expensive, noncurative therapy is approved, no consensus is often made among experts on ethical implications of societal cost. In the USA, third-party payers often require prior authorization to cover the cost of these treatments on individual basis. This is understandable since not all FDA approved drugs live up to the expectations in the postmarketing experience [6, 7]. The third-party payers rely on postmarketing report to approve the drug for routine treatment. Patient support groups are facing challenges to obtain payer approval for Ivacaftor in some countries. Dornase alpha which is now a standard CF therapy had faced a similar challenge when initially marketed [8]. It is estimated that there are only 1200 cystic fibrosis patients in the USA who would qualify for Ivacaftor therapy as it is currently approved. Although this new drug is rather expensive, prices of newly approved medications decline over time. Although our expectations for this treatment are optimistic we remain reserved in our predictions of application.

Rowe et al. conducted a longitudinal cohort study in CF patients with G551D mutations [4]. They assessed several parameters at 1, 3, and 6 months in 151 patients after starting Ivacaftor. They showed that there was significant and sustained improvement in BMI, pulmonary function tests, sweat chloride, cystic fibrosis respiratory symptom score, and mucociliary clearance. Their patients had a mean change of 6.7% predicted in their FEV1 value from baseline. The mean improvement in FEV1 in our patient group was 35%. The discrepancy between our study and Rowe et al.'s study is because of the following reasons. The mean FEV1 at baseline in our patient group was 50%, suggesting that our patients were much sicker with already established lung disease. On the contrary, patients studied by Rowe and coworkers were in a much healthier state with the mean FEV1 being 82.6% predicted. Since they already had their pulmonary functions in the normal range, they did not have much room to gain. All of our patients probably already had diffuse bronchiectasis to start with. They had more room to gain due to decrease in their mucus impaction relieving air flow obstruction as a consequence of Ivacaftor therapy. It is also notable that none of the pediatric patients in our group had mucoid* Pseudomonas* which may be another reason why they responded so well to Ivacaftor therapy.

An orphan drug such as Ivacaftor tends to be expensive due to smaller customer base and high cost of research and development. In Arkansas, three patients are suing the state's Medicaid [9] program for refusing to pay for Ivacaftor. Although clinicians cannot absolve themselves from the fiduciary responsibilities of health economics, their primary responsibility is to provide the best care for their own patients. At least in the United States, clinicians often have to petition third-party payers on behalf of their patients to get authorization for expensive therapies such as Ivacaftor. Since our study demonstrates that even in patients with established bronchiectasis there can be significant improvement in their pulmonary function, this information would be useful for other clinicians to successfully petition their third-party payers.

In our experience, all patients had significant both subjective and objective improvements in their health. Even in older patients with established lung disease there was improvement in FEV1. However, the effect was more dramatic in younger patients. It is known that the lungs are normal in CF patients when they are born and during their early part of life. However, mucus impaction, hypersecretion, changes in the mucus consistency, and chronic bacterial infection over time lead to irreversible lung damage. The only adult patient in our series who had CF related diabetes showed improved HbA1c% after Ivacaftor therapy. This is consistent with the observation made by Bellin et al. [10] who demonstrated improved insulin secretion in patients with CF related diabetes.

In order to have a greater impact on the long-term outcome, any novel therapy should be started quite early in life. Ivacaftor has not been approved for children under 6 years of age who are most likely to benefit from this drug. However, a clinical trial is underway in this age group and hopefully younger children will soon benefit from this novel therapy. To conclude, although Ivacaftor comes with a cost, it showed sustained improvement in FEV1, BMI, and functional status in all patients without any adverse effects. In our experience, Ivacaftor lives up to the expectation and brings about a new paradigm shift in current CF management.

## Figures and Tables

**Table 1:** Demography, radiology, and lower airway flora.

Patient/ age/gender	Mutation	CT scan before therapy	CT scan after therapy	Lower airway flora before Ivacaftor	Lower airway flora after Ivacaftor	Chronic antibiotic/ insulin
Number 1 14 yrs/F	del F508/G551D	6 months before: the multiple regions of scattered ground glass and tree in bud opacities in both lower lobes with scattered bronchiectasis predominantly in the upper lobes with mucoid impaction in distal airways	18 months after: the multiple regions of scattered ground glass and tree in bud opacities in both lower lobes and near resolution of the prior bibasilar ground glass and tree in bud opacities	MRSA∗	MRSA	None

Number 2 11 yrs/F	del F508/G551D	3 months before: diffuse bronchiectasis and peribronchial thickening in the left lower lobe	Not done	MRSA, NMPA^$^	MSSA^#^	None

Number 3 14 yrs/M	del F508/G551D	6 months before: peribronchial thickening and diffuse mild bronchiectasis	Not done	MRSA	Normal flora	None

Number 4 16 yrs/M	del F508/G551D	2 years before: diffuse bronchiectasis and mucoid impactions	6 months after: decreased mucoid impaction, improved bronchial wall thickening in comparison to CT scan 2 years ago	MRSA	MRSA	Inhaled tobramycin and colistin which was discontinued 1 year after Ivacaftor was started

Number 5 47 yrs/F	2789 + 5G > A/G551D	Not done	Not done	NMPA MPA^++^	NMPA MPA	Inhaled tobramycin Lispro-insulin

^*^MRSA: methicillin-resistant *Staphylococcus aureus*.

^#^MSSA: methicillin-sensitive *Staphylococcus aureus*.

^$^NMPA: nonmucoid *Pseudomonas aeruginosa*.

^++^MPA: mucoid *Pseudomonas aeruginosa*.

## Abbreviations

CFTR: Cystic fibrosis transmembrane conductance regulator
FEV1: Forced expiratory volume in 1 second
FEF 25–75: Forced expiratory flow between 25 and 75% of expiratory flow
BMI: Body mass index
FDA: Food and Drug Administration
CF: Cystic fibrosis
BAL: Bronchoalveolar lavage
MSSA: Methicillin-sensitive* Staphylococcus aureus*
MRSA: Methicillin-resistant* Staphylococcus aureus*
NMPA: Nonmucoid* Pseudomonas aeruginosa*
CT: Computed tomogram.
